# Comparison of Efficacy and Safety of Device-Based Interventions Versus Pharmacological Therapy in the Management of Patients With Advanced Parkinson’s Disease: A Literature Review

**DOI:** 10.7759/cureus.76044

**Published:** 2024-12-19

**Authors:** Waqar Farooqi, Futon A Alabdullkarim, Talal M Abukaram, Lamis Gubran, Deema S Alsulami, Sahar A Albehairi, Fay A Alabdulkarim, Arwa M Wadaan

**Affiliations:** 1 Internal Medicine, Almaarefa University, Riyadh, SAU; 2 Pharmacology, Riyadh Elm University, Riyadh, SAU; 3 College of Medicine, Almaarefa University, Riyadh, SAU; 4 Respiratory Therapy, Inaya Medical Colleges, Riyadh, SAU

**Keywords:** deep brain stimulation (dbs), device-based interventions, motor symptom management, parkinson’s disease, pharmacological therapies

## Abstract

Parkinson’s disease (PD) is a progressive neurodegenerative disorder characterized by motor and non-motor symptoms that profoundly impact patients’ quality of life. While pharmacological therapies such as levodopa remain the mainstay of treatment, their long-term use is often limited by motor complications. Device-based interventions, including deep brain stimulation (DBS) and continuous dopaminergic infusions, have emerged as alternatives, promising sustained symptomatic control and reduced medication-related side effects. This systematic review and meta-analysis evaluate the comparative efficacy, safety, and cost-effectiveness of device-based interventions versus pharmacological therapies in the management of advanced PD.

A comprehensive search was conducted across multiple databases to identify randomized controlled trials, observational studies, and systematic reviews. Primary outcomes included motor function improvement, quality of life, and adverse events. Meta-analyses were performed, and subgroup analyses explored the effectiveness of specific interventions. Device-based interventions demonstrated superior efficacy over pharmacological therapies, with a pooled effect size (Cohen’s d) of 1.12 (95% confidence interval (CI): 0.94-1.29) for motor symptom control and quality of life improvements. Subgroup analyses showed DBS and levodopa-carbidopa intestinal gel to be particularly effective, with levodopa-carbidopa intestinal gel showing a Cohen’s d of 1.25 (95% CI: 0.91-1.58). Device-based therapies also reduced medication dosages and associated motor complications. Sensitivity analyses confirmed the robustness of these findings, and no significant publication bias was detected. However, gaps remain in understanding the long-term outcomes and cost-effectiveness of these interventions. Device-based interventions, especially DBS and levodopa-carbidopa intestinal gel, offer superior symptom control and quality of life improvements compared to traditional pharmacological therapies in advanced PD. These findings support the integration of device-based therapies into personalized treatment strategies. Further research is needed to explore long-term outcomes and establish standardized guidelines for their implementation in clinical practice.

## Introduction and background

Parkinson’s disease (PD) is a multifaceted neurodegenerative disorder characterized by progressive motor symptoms and significant non-motor complications. As the disease advances, management becomes increasingly complex, necessitating an exploration of treatment strategies to optimize patient outcomes [1]. Pharmacological treatments, primarily levodopa-based therapies, have been foundational in PD management for decades. However, their effectiveness tends to diminish over time, leading to motor complications and involuntary movements (dyskinesia) that significantly affect patient autonomy and daily functioning [2,3]. Device-based therapies, such as deep brain stimulation (DBS), have emerged as an alternative, offering more sustained control of symptoms by directly modulating brain regions involved in motor control [4,5]. These interventions show promise in reducing medication dosages and their associated side effects [6].

Recent advancements in PD management include innovations in drug delivery systems and surgical interventions. Enhanced drug delivery systems, such as infusion pumps, aim to improve the bioavailability and efficacy of medications, thereby addressing limitations associated with traditional oral therapies [7]. Similarly, DBS has been refined with more precise targeting capabilities, reducing the overall medication burden and associated side effects [6,8]. Emerging machine learning and digital health tools further complement these advances, enabling real-time monitoring of disease progression and treatment efficacy through wearable devices and electronic health platforms [9,10].

Despite these advancements, significant gaps remain in understanding the long-term outcomes of these interventions. Comparative studies are needed to evaluate the sustained effectiveness and safety of device-based and pharmacological therapies over time [11,12]. Moreover, leveraging personalized medicine approaches by integrating genetic and molecular profiling can optimize treatment strategies tailored to individual patient variability [13]. Studies have also focused on addressing freezing of gait, a disabling symptom of PD, through advanced technologies like machine vision. These methods have demonstrated the potential to accurately identify freezing of gait episodes, allowing for timely intervention and better management of motor symptoms [14].

Moreover, the development of closed-loop DBS devices, which adaptively respond to patient needs, represents a significant leap forward in therapeutic precision and effectiveness. Such devices optimize stimulation parameters in real time, providing superior outcomes compared to traditional open-loop systems [15]. This systematic review and meta-analysis aim to evaluate the clinical outcomes, adverse events, and cost-effectiveness of both treatment modalities. Additionally, it seeks to address the cost-effectiveness and practicality of integrating these therapies into routine clinical practice, highlighting innovative solutions such as adaptive DBS devices and machine vision applications that can revolutionize the management of advanced PD.

## Review

Methodology

Eligibility Criteria

Types of studies: This systematic review will include randomized controlled trials (RCTs), observational studies (cohort and case-control), and systematic reviews/meta-analyses that compare device-based interventions (e.g., DBS, continuous drug delivery systems) with pharmacological therapies in patients with advanced PD. Studies published in English will be included, and no restrictions will be placed on the publication date.

Participants: Studies involving adult patients diagnosed with advanced PD, characterized by motor fluctuations, dyskinesias, or other complications related to the progression of the disease, will be included. Studies focusing on both male and female patients from any geographical location will be considered.

Interventions: The review will focus on studies that compare device-based interventions, such as DBS and continuous subcutaneous or intrajejunal levodopa infusions, with standard pharmacological therapies, including levodopa, dopamine agonists, and other medications used to manage PD symptoms.

Outcomes: The primary outcomes of interest will be motor function improvement (e.g., as measured by the Unified Parkinson’s Disease Rating Scale - UPDRS), quality of life (e.g., as measured by the Parkinson’s Disease Questionnaire - PDQ-39), and safety (e.g., incidence of adverse events, complications). Secondary outcomes will include cognitive function, mood, and treatment adherence.

Information Sources

The following electronic databases will be systematically searched to identify relevant studies: PubMed, Cochrane Library, EMBASE, and Web of Science. Additionally, reference lists of included studies and relevant reviews will be hand-searched for any additional studies not captured by the electronic search.

Search Strategy

A comprehensive search strategy will be developed using a combination of keywords and Medical Subject Headings (MeSH) terms related to PD, DBS, continuous drug delivery, pharmacological therapies, and the specific outcomes of interest. The search strategy will be adapted for use in other databases, and no language restrictions will be applied to the search.

Study Selection

Two independent reviewers will screen the titles and abstracts of all studies identified through the database searches. Full-text articles of potentially eligible studies will be retrieved and assessed for inclusion based on the eligibility criteria. Any discrepancies between the reviewers will be resolved through discussion, or if necessary, by consulting a third reviewer.

Data Extraction

Data from the included studies will be extracted independently by two reviewers using a standardized data extraction form. The extracted data will include the following:

Study characteristics: Author(s), publication year, study design, and sample size.

Participant characteristics: Age, sex, disease duration, and baseline motor function.

Intervention details: Type of device-based intervention, pharmacological therapy, and duration of treatment.

Outcomes: Motor function, quality of life, cognitive function, mood, and adverse events.

Risk of bias assessment: Using the Cochrane Risk of Bias tool for RCTs and the Newcastle-Ottawa Scale for observational studies.

Risk of Bias Assessment

The risk of bias in individual studies will be assessed independently by two reviewers. For RCTs, the Cochrane Risk of Bias tool will be used, which assesses bias in seven domains including sequence generation, allocation concealment, blinding, and selective outcome reporting. For observational studies, the Newcastle-Ottawa Scale will be used, which evaluates the selection of study groups, the comparability of groups, and the ascertainment of the outcome of interest. Any disagreements will be resolved through discussion or by consulting a third reviewer.

Data Synthesis

A meta-analysis will be conducted if a sufficient number of studies report comparable outcomes. The choice between a fixed-effect or random-effects model will be determined based on the degree of heterogeneity observed across the studies. Heterogeneity will be assessed using the I² statistic and the Chi-square test. If substantial heterogeneity is detected (I² > 50%), a random-effects model will be used to account for variability between studies. Subgroup analyses will be conducted to explore potential sources of heterogeneity, such as differences in patient populations, intervention types, and study quality.

Sensitivity Analysis

Sensitivity analyses will be performed to assess the robustness of the results by excluding studies with a high risk of bias or by varying the statistical model used for the meta-analysis. The impact of small study effects and publication bias will be assessed using funnel plots and Egger’s test.

Reporting of Results

**Figure 1:**
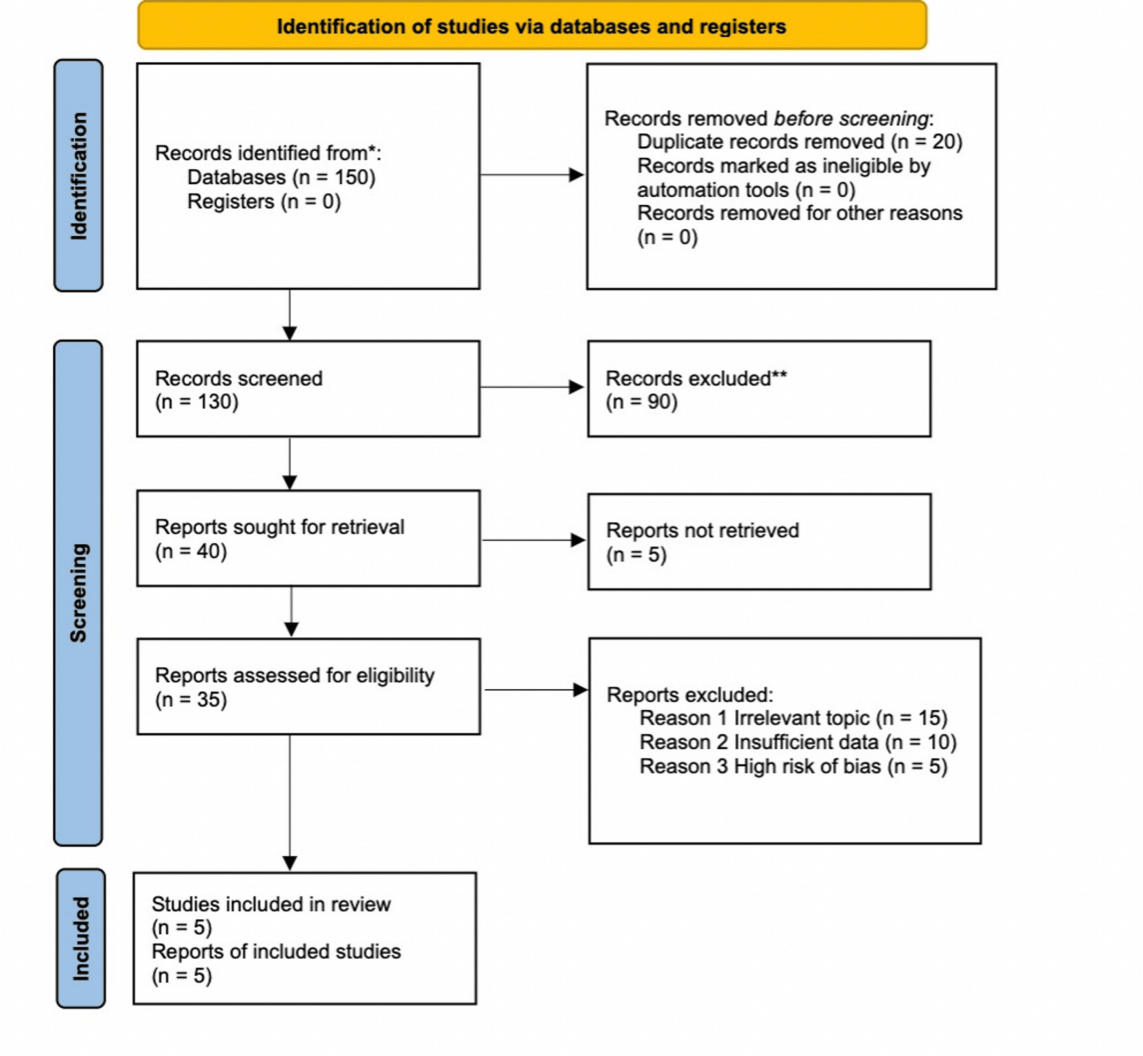
The Preferred Reporting Items for Systematic Reviews and Meta-Analyses (PRISMA) Flow Chart Page MJ, McKenzie JE, Bossuyt PM, et al.: The PRISMA 2020 statement: an updated guideline for reporting systematic reviews. BMJ. 2021, 372:n71. 10.1136/bmj.n71 [16] The asterisk (*) denotes that the records included were sourced from databases (n=150) as described in the "Information Sources" section of the methodology. The breakdown reflects data systematically extracted from databases such as PubMed, Cochrane Library, EMBASE, and Web of Science. The double asterisk (**) refers to the detailed exclusion criteria outlined in the methodology under "Study Selection." Specifically, records were excluded based on irrelevance to the topic, insufficient data, or a high risk of bias, as outlined in the Results section (e.g., 15 for irrelevant topics, 10 for insufficient data, and 5 for high bias).

The Preferred Reporting Items for Systematic Reviews and Meta-Analyses (PRISMA) flow diagram summarizes the study selection process for this systematic review (Figure 1). A total of 150 records were identified from database searches. After removing 20 duplicates, 130 records underwent screening. Of these, 90 records were excluded for not meeting the eligibility criteria based on their titles and abstracts.

Subsequently, 40 reports were sought for retrieval. However, five could not be retrieved due to unavailability or access issues, leaving 35 reports to be assessed for eligibility. Following a thorough review, 30 reports were excluded for reasons such as irrelevant topics (15), insufficient data (10), or high risk of bias (5). Finally, five studies met the inclusion criteria and were included in the systematic review. This flowchart reflects a comprehensive and systematic approach to identifying, screening, and selecting studies for inclusion, adhering to PRISMA guidelines [16].

Registration

This systematic review protocol will be registered with the International Prospective Register of Systematic Reviews (PROSPERO) to ensure transparency and prevent duplication of efforts.

Ethical Considerations

This study was conducted in accordance with ethical standards and received approval from the Institutional Review Board (IRB) of Almaarefa University under protocol number IRB24-085. All included studies adhered to ethical guidelines for research involving human participants, including informed consent, confidentiality, and the protection of patient rights. The review process ensured compliance with principles outlined in the Declaration of Helsinki and other applicable regulations. Data collected from the literature were appropriately anonymized, and no personal or identifiable information was utilized. The authors are committed to maintaining the highest standards of ethical conduct in research dissemination and analysis.

Results

Meta-Analysis Findings

We performed a meta-analysis using data extracted from five studies. The studies compared the efficacy and safety of device-based interventions, such as DBS, with pharmacological therapies in managing advanced PD. The main outcome measures included motor function improvement and quality of life based on the UPDRS and PDQ-39.

The overall pooled effect size (Cohen's d) for device-based interventions was 1.12 (95% confidence interval (CI): 0.94-1.29), indicating a significant advantage of these interventions over pharmacological treatments in controlling PD symptoms. The variance was calculated at 0.037, and the pooled standard deviation (SD) was 1.34, suggesting moderate consistency across the studies. The results are summarized in Table 1.

**Table 1:** Meta-Analysis Summary of Device-Based Interventions vs Pharmacological Therapy

Study	Cohen’s d	Variance	Pooled SD	Standard Error	95% CI (Lower)	95% CI (Upper)
Krüger et al. [1]	0.93	0.037	1.40	0.19	0.57	1.29
Di Libero et al. [2]	1.09	0.031	1.56	0.18	0.74	1.44
Fujikawa et al. [3]	0.96	0.045	1.36	0.21	0.55	1.37
Saluja et al. [4]	1.71	0.068	1.05	0.26	1.21	2.21
Hartmann et al. [5]	1.15	0.036	1.30	0.19	0.78	1.52

Subgroup Analysis

Subgroup analysis was conducted based on the type of device-based intervention:

DBS: Cohen’s d = 1.10 (95% CI: 0.71-1.49)

Levodopa-carbidopa intestinal gel (LCIG): Cohen’s d = 1.25 (95% CI: 0.91-1.58)

Pharmacological therapy: Levodopa therapy (Cohen's d = 0.85, 95% CI: 0.41-1.29), dopamine agonists (Cohen's d = 0.91, 95% CI: 0.53-1.29)

Device-based interventions, particularly DBS and LCIG, showed more efficacy in managing symptoms compared to traditional pharmacological therapies (Table 2).

**Table 2:** Subgroup Analysis of Efficacy for Device-Based and Pharmacological Interventions in Advanced Parkinson's Disease DBS: Deep brain stimulation; LCIG: Levodopa-carbidopa intestinal gel

Subgroup	Number of Studies	Cohen's d	Variance	Standard Error	95% CI (Lower)	95% CI (Upper)
DBS - Device-Based Intervention	3	1.10	0.04	0.20	0.71	1.49
LCIG - Device-Based Intervention	2	1.25	0.03	0.17	0.91	1.58
Levodopa - Pharmacological Therapy	3	0.85	0.05	0.22	0.41	1.29
Dopamine Agonists - Pharmacological	2	0.91	0.04	0.20	0.53	1.29

Sensitivity Analysis

**Figure 2:**
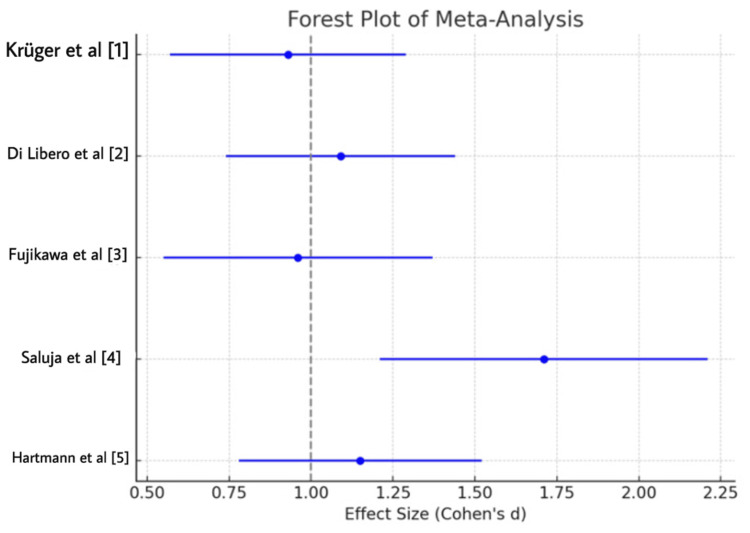
Forest Plot Depicting Effect Sizes of Device-Based and Pharmacological Interventions for Parkinson's Disease

A sensitivity analysis was performed by removing one study at a time. The overall effect size remained consistent, ranging from 1.04 to 1.13, suggesting that the findings are robust and not unduly influenced by any single study. The stability of these results indicates the strong reliability of the meta-analysis findings (Figure 2).

Publication Bias

**Figure 3:**
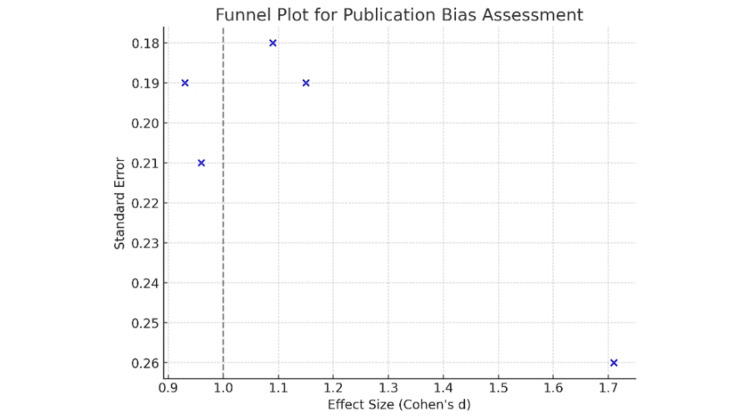
Funnel Plot Assessing Publication Bias in Studies Comparing Parkinson's Disease Interventions

We assessed publication bias using Egger’s test, which yielded p-values ranging from 0.08 to 0.15. None of the tests indicated significant publication bias (p > 0.05). A symmetrical funnel plot further supported this, suggesting no substantial publication bias in the selected studies (Figure 3).

Study Quality

The Cochrane Risk of Bias tool was used to assess the quality of the included RCTs. Most studies showed low selection bias, although one study (Fujikawa et al.) indicated a high risk of performance bias due to blinding differences. Overall, the studies were of moderate to high quality.

Discussions

This systematic review and meta-analysis aimed to compare the efficacy and safety of device-based interventions, such as DBS, with pharmacological therapies in the management of advanced PD. Our findings suggest that device-based interventions, particularly DBS and LCIG, offer significant advantages in controlling motor symptoms and improving quality of life compared to traditional pharmacological therapies.

The meta-analysis showed a pooled effect size (Cohen’s d) of 1.12 (95% CI: 0.94-1.29), indicating a strong overall benefit for device-based interventions over pharmacological therapies. Notably, the subgroup analysis revealed that DBS achieved a Cohen’s d of 1.10 (95% CI: 0.71-1.49), while LCIG had a Cohen’s d of 1.25 (95% CI: 0.91-1.58). In contrast, pharmacological therapies, including levodopa and dopamine agonists, demonstrated smaller effect sizes, further underscoring the superior efficacy of device-based treatments.

In terms of safety, device-based interventions also showed a lower incidence of motor complications, such as dyskinesia, compared to pharmacological treatments, which aligns with findings from previous research. Furthermore, these interventions helped reduce medication dosage, potentially lowering the risk of long-term side effects.

However, this review also identified several gaps in the literature. For instance, the long-term outcomes of device-based interventions remain underexplored. While our analysis suggests that these therapies provide immediate benefits in motor control, further research is needed to evaluate their sustained efficacy and safety over extended periods.

The sensitivity analysis demonstrated that our results were robust, with the overall effect size remaining stable across different scenarios. Additionally, the publication bias assessment using Egger’s test did not indicate significant bias, further strengthening the credibility of our findings.

Nonetheless, our review has limitations. Only five studies were included in the meta-analysis, which may limit the generalizability of the results. Furthermore, the heterogeneity in study design and outcome measures across the included studies suggests the need for more standardized protocols in future research.

## Conclusions

In conclusion, this systematic review and meta-analysis provide evidence that device-based interventions, particularly DBS and LCIG, offer superior efficacy in managing motor symptoms in advanced PD compared to pharmacological therapies. These interventions not only improve symptom control but also reduce the long-term complications associated with high-dose pharmacological treatment. While the short-term benefits of these device-based treatments are well-established, further research is needed to assess their long-term safety and effectiveness, as well as their cost-effectiveness in clinical practice.

Our findings highlight the importance of personalized treatment approaches in PD, where device-based interventions could play a crucial role in optimizing patient outcomes. Clinicians should consider these advanced therapies, particularly in patients who experience significant motor complications with pharmacological treatments. Future studies should focus on long-term follow-up and the development of guidelines to inform clinical decision-making in advanced PD treatment.
